# The evaluation of IMERG and ERA5-Land daily precipitation over China with considering the influence of gauge data bias

**DOI:** 10.1038/s41598-022-12307-0

**Published:** 2022-05-16

**Authors:** Wenhao Xie, Shanzhen Yi, Chuang Leng, Defeng Xia, Mingli Li, Zewen Zhong, Jianfeng Ye

**Affiliations:** 1 Key Laboratory of Digital Watershed of Hubei Province, School of Civil and Hydraulic Engineering, Huazhong University of Science and Technology, Wuhan, 430074 China; 2 Center for Operation and Management on Watershed Hub, China Three Gorges Corporation, Yichang, 443134 China

**Keywords:** Climate sciences, Environmental sciences, Hydrology

## Abstract

Evaluating the accuracy of the satellite and reanalysis precipitation products is very important for understanding their uncertainties and potential applications. However, because of underestimation existing in commonly used evaluation benchmark, gauge precipitation data, it is necessary to investigate the influence of systematic errors in gauge data on the performance evaluation of satellite and reanalysis precipitation datasets. Daily satellite-based IMERG and model-based ERA5-Land, together with gauge precipitation data, were collected with the period from 2005 to 2016 over China in this study. Daily corrections for precipitation biases from wind-induced undercatch, wetting loss, and trace error were made for gauge measurements. A set of metrics, including relative bias, Kling-Gupta efficiency, frequency bias, and critical success index, were used to evaluate and intercompare the performances of IMERG and ERA5-Land against original and bias-corrected gauge data in different locations, years, seasons, climatic zones, classes of precipitation events, and precipitation phases. The results have shown that: After removing the bias in gauge data, the relative biases of IMERG and ERA5-Land both significantly decline. The noticeable changes of their accuracy occur and vary with different locations, years, seasons, climatic zones, and precipitation phases. Furthermore, the frequency biases of IMERG and ERA5-Land rise in no precipitation events and decline in light, moderate, heavy, and extreme precipitation events. The detection capability of IMERG and ERA5-Land in no and light precipitation events is also obviously affected. Therefore, this study has demonstrated the significant influence of systematic gauge precipitation errors on the assessment of IMERG and ERA5-Land and reinforces the necessity to remove negative bias in gauge data before using it as the benchmark.

## Introduction

Accurate and reliable information on precipitation is of great importance in water resources planning, hydrologic simulation, environmental and ecological management, drought monitoring and irrigation management, and other applications^1–9^. At present, there are three approaches to obtain precipitation observation or estimation data, namely^10^: (i) in situ measurements, (ii) remote sensing (including ground-based weather radar and satellite), and (iii) numerical simulation.

Rain gauge is the most common and reliable way to directly measure precipitation at point scale^11^. However, the density and distribution of the rain gauge networks vary significantly across the globe, with relatively dense networks of gauge stations in developed counties but scarce or even no gauge stations over marine, mountainous areas, and developing countries^12^, making it challenging to offer continuous precipitation information.

By measuring reflected and emitted radiation at a distance, remote sensing can indirectly acquire information about precipitation without making physical contact with hydrometeors. Due to the ability to detect precipitation across wide spatial coverage in all weather conditions, satellite-based methods become major remote sensing approaches to collect continuous precipitation over large areas. Until now, a global meteorological satellite network has been established through international cooperation and a huge amount of data has been accumulated. Numerical model simulation is another important way to obtain spatially and temporally continuous precipitation information at global and regional scales. Reanalysis, a scientific method assimilating historical atmospheric observational data with outputs from numeric weather prediction models, produces improved quality field data spanning an extended period compared to individual data sources.

Due to the ability to provide global and continuous coverage, the satellite and reanalysis precipitation products provide an unprecedented opportunity to overcome the disadvantage of rain gauges distributing spatially discretely and have shown great potential in a wide range of applications^13–16^.

However, the satellite and reanalysis precipitation products are derived from indirect precipitation measure and simulation. The errors of the products exist and vary in different regions, seasons, classes of precipitation events, and precipitation phases. Evaluating satellite and reanalysis precipitation products is an important activity for applications. The related studies for this issue have been performed to investigate and evaluate satellite and reanalysis precipitation products with respect to rain-gauge data in China^17–24^. For example, Deng et al. compared gridded monthly satellite-based CMORPH and reanalysis-based NCEP-2 at seasonal and regional scales over China^18^. They found that NCEP-2 has a larger error over southern China in the summer and CMORPH has poor consistency with gauge precipitation observations over western and northwestern China during all seasons. Yang et al. evaluated the performance of three satellite-based IMERG products (Early Run, Late Run, and Final Run) in detecting general, heavy, and extreme rainfall events^22^. The results show that IMERG products have much better detection ability in general rainfall events than in heavy and extreme rainfall events. Tang et al. used the triple collocation analysis to evaluate the snowfall accuracy of IMERG, two reanalysis products (ERA5 and ERA5-Interim), and gauge observations^20^. The results suggest that IMERG snowfall is worse than reanalysis and gauge data. The previous studies have provided valuable information on the estimation error of satellite and reanalysis precipitation products, which is of great importance for the users to select appropriate satellite and reanalysis precipitation products for different applications. Nonetheless, all of them were conducted using gauge precipitation data from direct observation which has been reported to underestimate actual precipitation^25–28^.

The biases of gauge precipitation measurements include trace precipitation, wetting losses, evaporating losses, and wind undercatch^26^. The underestimation of observation from gauge could bias the gauge measurements and weaken the reliability of the assessment results of satellite and reanalysis estimates^29–31^. Thus, it is important to survey the impact of the systematic underestimation in the gauge precipitation data on the evaluation of satellite and reanalysis products using a bias-corrected version of gauge data. In China, study on bias correction of gauge data has been widely conducted since the 1980s, and several gauge-measured precipitation bias correction methods were obtained and applied^32–37^. For example, according to the World Meteorology Organization (WMO) gauge intercomparison of precipitation measurements, Yang et al. has studied the accuracy of observed precipitation from Chinese standard precipitation gauge (CSPG) and established the relationship between wind-caused error and wind speed, precipitation type, and precipitation rate^38^.

In this study, IMERG (Integrated Multi-satellitE Retrievals for Global Precipitation Measurement (GPM))^39^ and ERA5-Land (the new land component of the fifth generation of European ReAnalysis (ERA5))^40^ were selected to be evaluated. IMERG was chosen because of its state-of-art multi-satellite fusion algorithm and relatively better performance over China than other satellite precipitation products. From the beginning of the release of IMERG, the algorithm is constantly updated and the latest version is V06B developed in 2019. Tang et al.^20^ compared IMERG and six other satellite-based products (TRMM 3B42, CMORPH, PERSIANN-CDR, GSMaP, CHIRPS, and SM2RAIN) at daily and hourly scales in China. The results showed that the IMERG product outperforms other datasets, except GSMaP, which uses daily-scale station data to adjust satellite precipitation estimates. ERA5-Land was chosen due to its high spatial and temporal resolution (9 km and hourly), the use of the state-of-art land surface modeling (H-TESSEL), and sharing advanced 4D-var data assimilation scheme and numerical weather prediction model with ERA5^40^. ERA5-Land is produced through global high-resolution numerical integrations of the ECMWF land surface model driven by the downscaled meteorological forcing from the ERA5 climate reanalysis, including an elevation correction for the thermodynamic near-surface state. Compared to ERA5, ERA5-Land has an enhanced horizontal resolution, which is 9 km compared to 31 km (ERA5), making it useful for diverse applications dealing with water resource, land, and environmental management.

This study aims to investigate the influence of systematic bias in gauge data on the evaluation of IMERG and ERA5-Land in different locations, years, seasons, climatic zones, classes of precipitation events, and precipitation phases. To do this, we first remove the negative bias inherent in gauge observations in China. Then, we present and inter-compare the evaluation results of IMERG and ERA5-Land using original and bias-corrected gauge measurements, respectively. The structure of this study is as follows: “Materials and methods” section describes the study area, datasets, and methods used in this study. “Results” section presents the comprehensive evaluation results. “Discussion” section makes the discussion. Finally, “Conclusion” section concludes the paper.

## Materials and methods

### Study area

**Figure 1:**
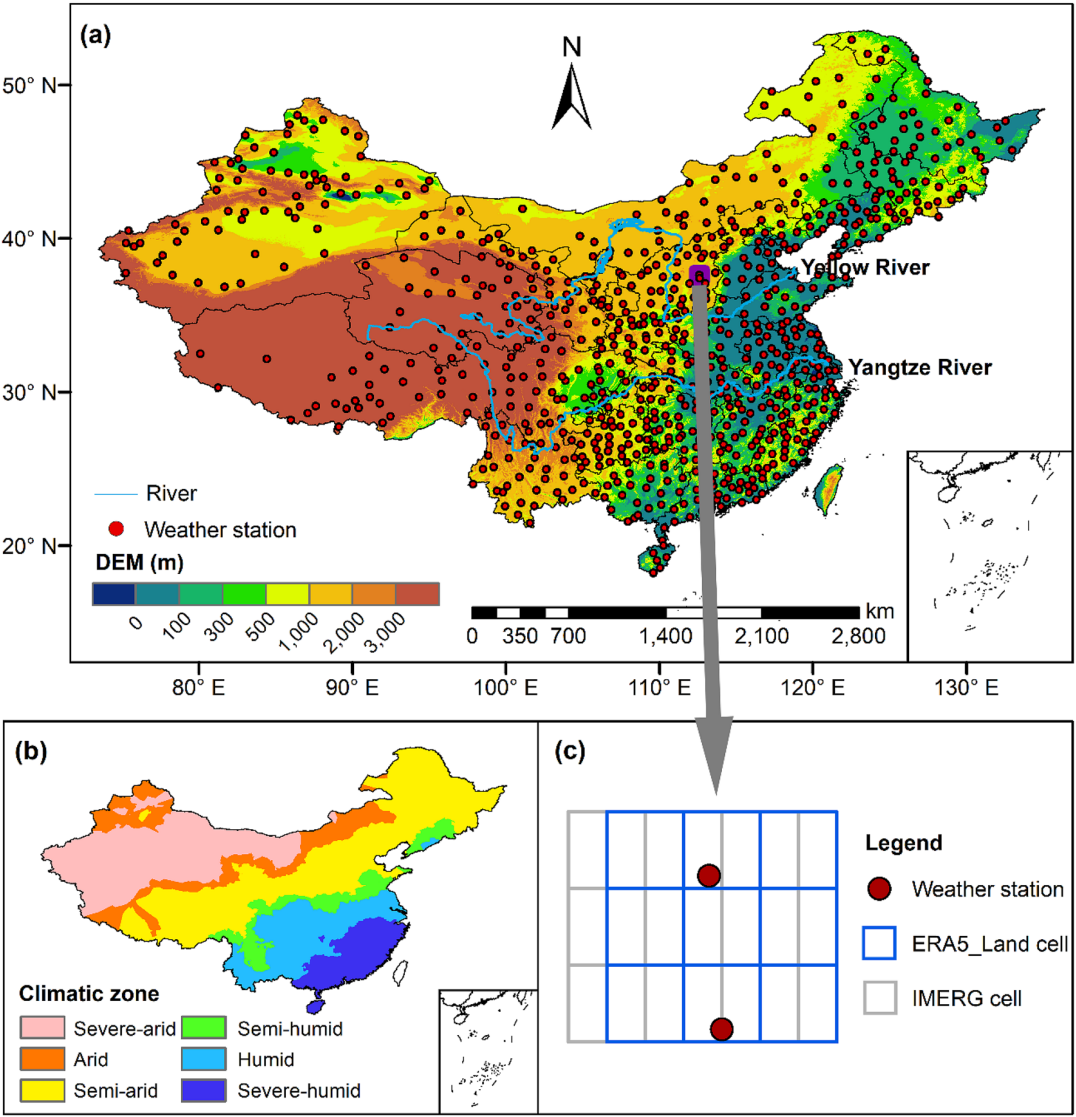
(**a**) Topography and the location of the selected weather stations over China. (**b**) Climatic zones using multi-year average precipitation from bias-corrected gauge data (see Table 1). (**c**) Enlarged detail plot of two nearest weather stations along with ERA5_Land cell and IMERG cell for the purple area in (**a**). All of the maps in this figure and Figs. 2, 3, 4 and 5 were created by ArcGIS 10.2 software (https://resources.arcgis.com/en/help/main/10.2/).

The study area, China, has a descending elevation ranging from west to east (Fig. 1a), with much diverse topography, including plains, valleys, hills, mountainous, river systems, water bodies, plateau, desert, glacier, etc^41^. In China, precipitation variation in space and time is mainly affected by monsoon winds^42–45^. Summer monsoons including southwest and southeast monsoons from the equatorial Indian Ocean and the western Pacific Ocean transport warm and humid air into the Chinese mainland and produce the majority of summer precipitation in China. Nevertheless, the Siberian anticyclone dominates in winter and brings cold and dry weather conditions to much of China. The rainy season mainly occurs from May to September. Southern China starts the rainy season in April and May, emerges persistently overcast and rainy weather phenomenon called plum rain in June and July, and ends rainy season in October. Northern China begins the rainy season in July and August, ending in September.

**Table 1:** The division of climatic zones using annual mean precipitation based on bias-corrected gauge data.

Climatic zone	Annual mean precipitation (mm/year)
Severe-arid	< 250
Arid	250–400
Semi-arid	400–800
Semi-humid	800–1000
Humid	1000–1600
Severe-humid	≥ 1600

**Figure 2:**
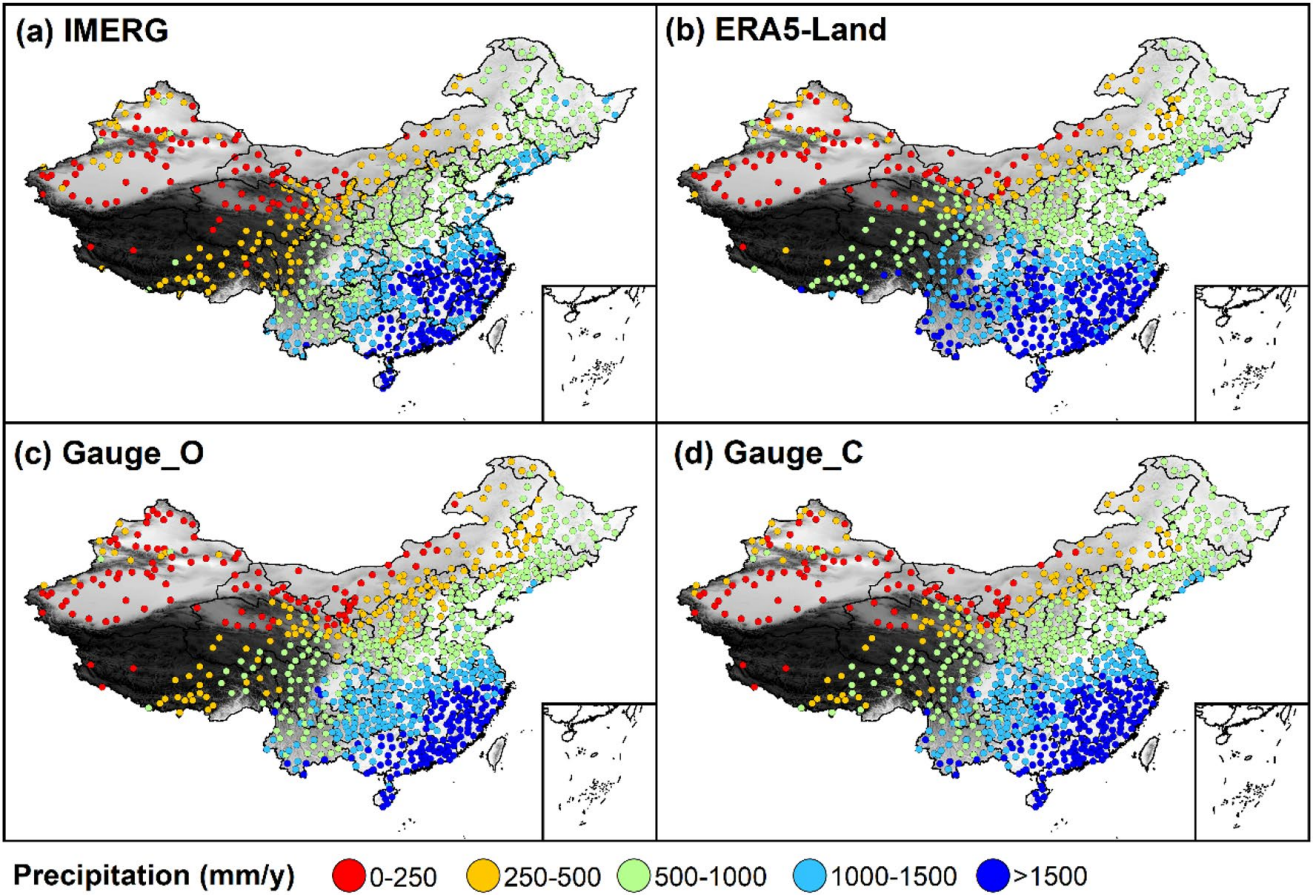
Spatial distribution of annual mean precipitation over China from (**a**) IMERG, (**b**) ERA5-Land, (**c**) Gauge_O, and (**d**) Gauge_C.

**Figure 3:**
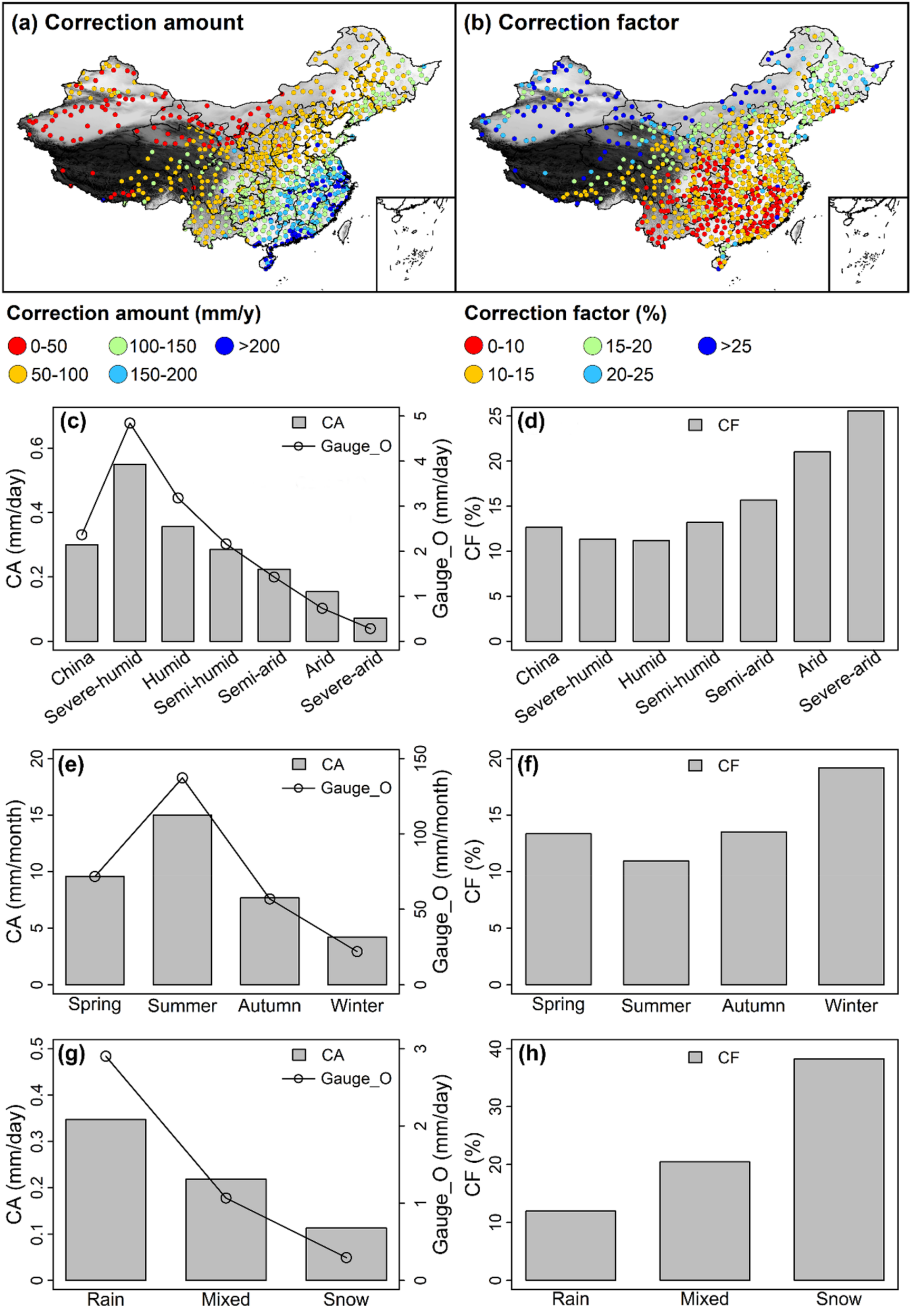
The correction amount (CA) and correction factor (CF) in different locations (**a,b**), climatic zones (**c,d**), seasons (**e,f**), and precipitation phases (**g,h**).

**Figure 4:**
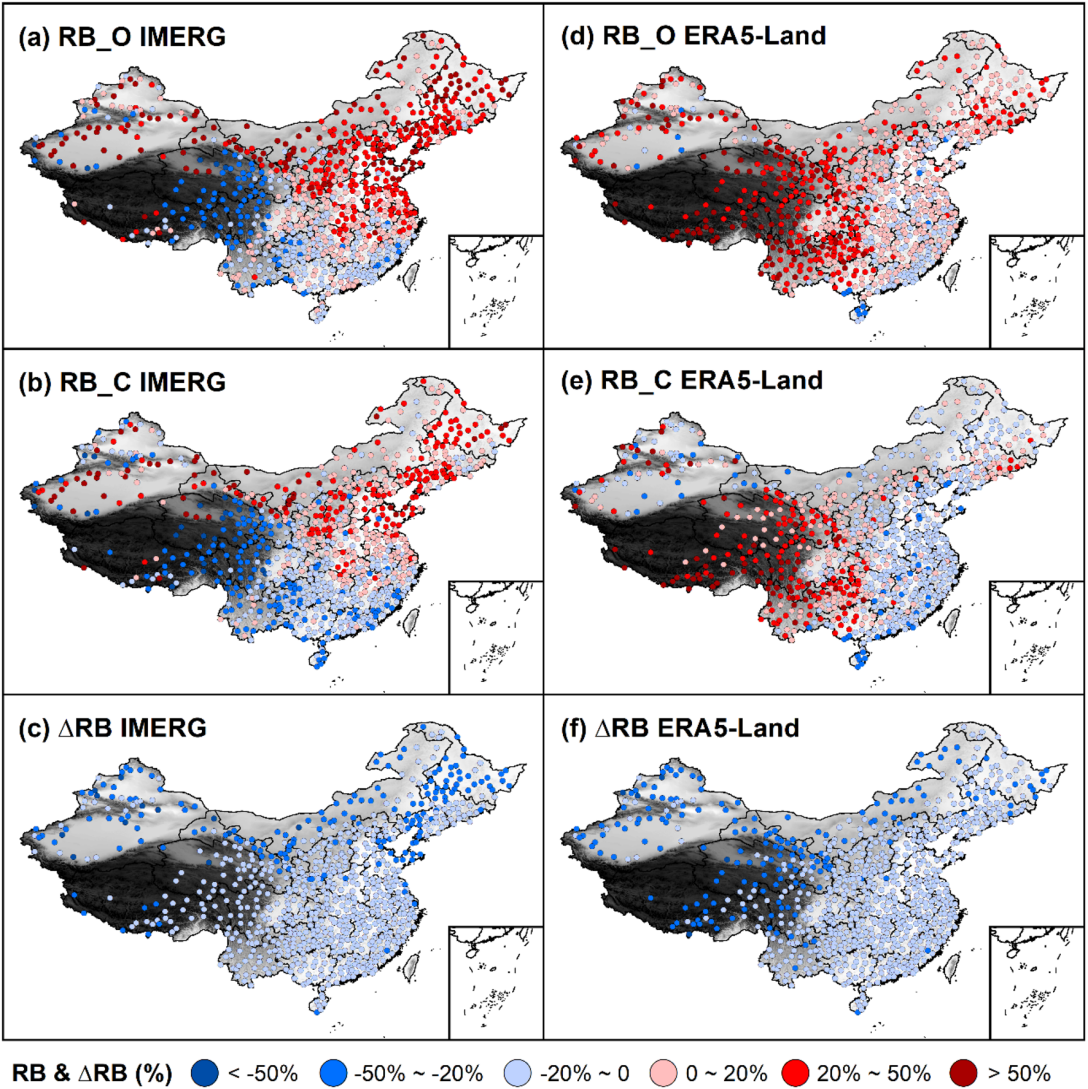
Spatial distributions of RB_O, RB_C, and ∆RB for IMERG and ERA5-Land.

**Figure 5:**
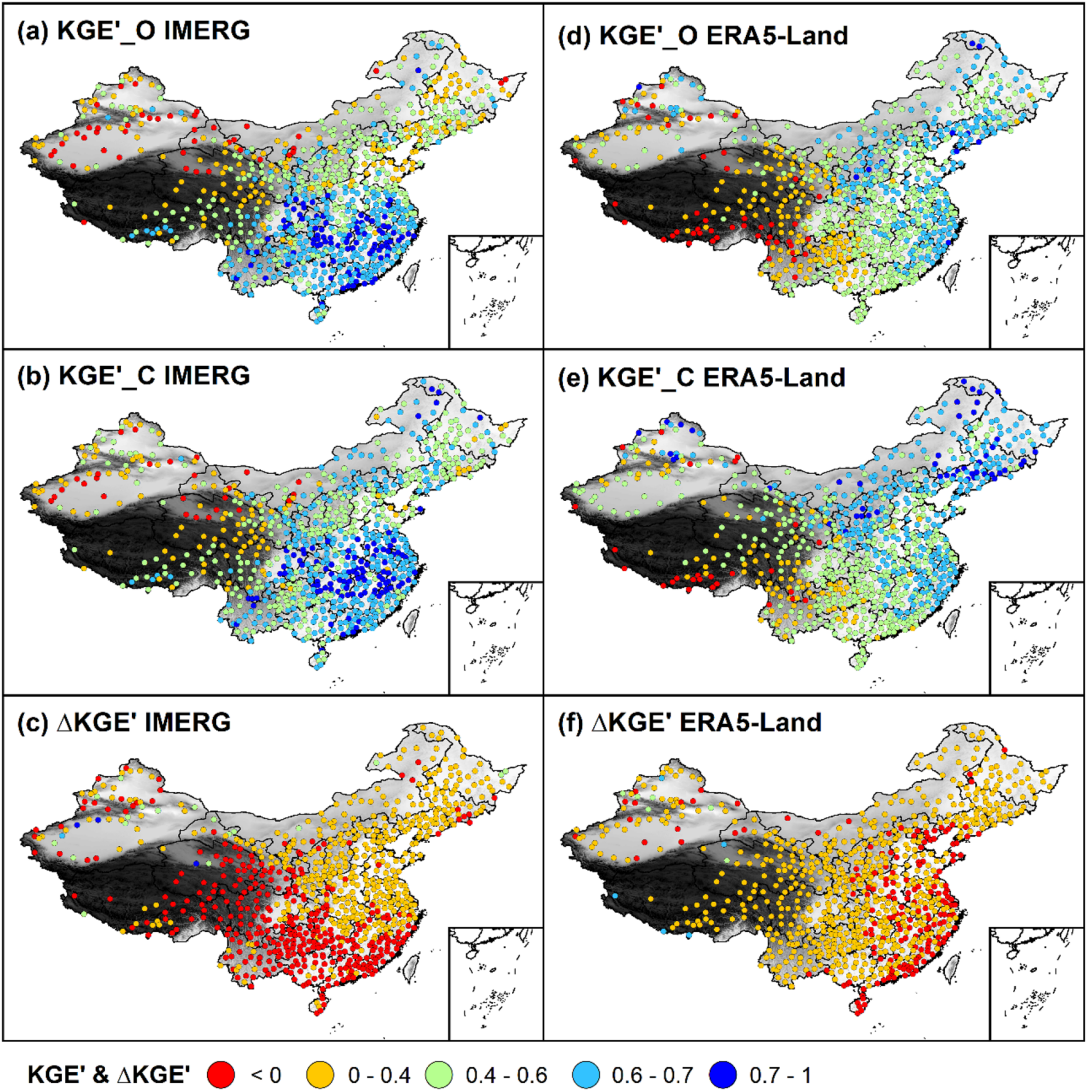
Spatial distributions of KGEʹ_O, KGEʹ_C, and ∆KGEʹ for IMERG and ERA5-Land.

According to the multi-year average precipitation of China, the study area could be divided into several precipitation climatic zones^46^, as shown in Fig. 1b. The division of climatic zones using annual mean precipitation with bias-corrected gauge data is shown in Table 1.

### Data sets

#### Gauge data

Twelve years (2005–2016) of the daily gauge precipitation data with strict quality control and inspection from 814 weather stations (Fig. 1a) operated by the China Meteorological Administration (CMA) were obtained. CMA stations use the Chinese standard precipitation gauge (CSPG) as the standard instrument for measuring both solid and liquid precipitation since the late 1950s. CSPG is a cylinder of galvanized iron, 65 cm long and 20 cm in diameter and is placed 0.7 m above the ground without a windshield. CSPG is used with a funnel and a glass bottle for rain measurements. For solid measurements, the funnel and the glass bottle are removed. Precipitation is measured twice a day over China, at 0800 and 2000 LT (local, Beijing, time). Besides, the daily mean air temperature, relative humidity, air pressure, station elevation, and daily mean wind speed at 10 m height from these CMA stations were also gathered, which will be used for the distinguishing of precipitation phases (i.e., rainfall, snowfall, and mixed precipitation) and the removing of systematic bias in gauge precipitation data. As shown in Fig. 1a, the distribution of weather stations is sparse (the nearest great circle distance between stations is 22.25 km (Fig. 1c)) and uneven (the weather stations are denser in eastern China than in western China). In this study, original and bias-corrected gauge precipitation data are labeled as Gauge_O and Gauge_C.

#### IMERG

As one of the most popular algorithms of GPM, IMERG intercalibrates, merges, and interpolates microwave precipitation estimates from GPM constellation, microwave-calibrated infrared (IR) satellite estimates, Global Precipitation Climatology Centre (GPCC) monthly gauge analyses, and data from other sensors for the TRMM and GPM eras^39^. The latest Version 06B of IMERG extends the time range of data back to June 2000 by integrating the precipitation estimates collected during the TRMM era. The system runs three times for each observation time, providing Early Run (near real-time), Late Run (near real-time), and Final Run (research-level) products. The IMERG Final Run product includes two precipitation variables: the microwave-infrared estimates without gauge calibration (IMERG_uncal) and the calibrated product based on GPCC monthly gauge analysis (IMERG_cal). Because the gauge observations from weather stations used in this study are incorporated in GPCC monthly gauge analysis but not assimilated into ERA5-Land, the IMERG_uncal (hereafter IMERG) rather than IMERG_cal is selected for an independent and fair evaluation. The half-hourly 0.1° IMERG Final Run precipitation estimates were gathered from Goddard Earth Sciences Data and Information Services Center (GES DISC) (https://disc.gsfc.nasa.gov/).

#### ERA5-Land

ERA5-Land, produced by replaying the land component of the ECMWF ERA5 climate reanalysis, offers finer-scale resolution (9 km) and more accurate land parameters than 31 km ERA5-HRES. Another critical difference between the two products is that ERA5-Land is only forced by the ERA5 low atmosphere meteorological field in conjunction with an additional lapse-rate correction and is not coupled to the atmospheric model, which is designed to make running ERA5‑Land computationally affordable^47^. The precipitation data used in this paper is the sum of large-scale and convective precipitation from the beginning to the end of the forecast step. The regular hourly ERA5-Land precipitation estimates with 0.1° × 0.1° latitude/longitude grid were downloaded from the Climate Data Store (CDS) (https://cds.climate.copernicus.eu/).

### Methods

#### Pre-processing of the datasets

It should be noted that CMA records daily precipitation from 8:00 p.m. Beijing time of the previous day to 8:00 p.m. Beijing time of the current day, which is equivalent to 12:00 Coordinated Universal Time (UTC) to 12:00 UTC. To get the consistent definition of daily precipitation with CMA, we aggregated all half-hourly IMERG and hourly ERA5-Land estimates falling between 12:00 and 12:00 UTC to get daily IMERG and ERA5-Land precipitation.

Due to point measurement characteristic, gauge data do not match the areal estimates of IMERG and ERA5-Land^48^. To deal with this issue, multiple rain gauge observations within a pixel can be averaged to approximate areal precipitation in the presence of dense gauge networks. However, the gauge network used in this study is sparse. The fact is that the nearest great circle distance between stations is 22.25 km, which is larger than one grid distance of IMERG and ERA5-Land (Fig. 1c). As alternative solutions, different interpolation methods can be used to downscale areal estimates of IMERG and ERA5-Land to point scale or upscale point measurement of gauge to areal precipitation. However, these methods vary with different hydroclimate and terrain and the uncertainty introduced by interpolation itself is hard to quantify.

Therefore, to preserve the accuracy of the rain gauge data, IMERG and ERA5-Land, we adopted a pixel-to-point strategy adopted by many other authors in data-scarce regions^21,49–53^ to evaluate IMERG and ERA5-Land. This strategy neglects the scale gap between daily precipitation estimates from gridded IMERG and ERA5-Land and their counterpart derived from rain gauge point measurement and assumes the grid-scale precipitation is equal to point-scale precipitation regardless of the gauge position in the pixel. Precipitation from the specific pixels of IMERG and ERA5-Land were extracted using the geographic location of weather stations and were compared with the corresponding gauge observations by the following metrics.

#### Evaluation metrics

To assess the accuracy of IMERG and ERA5-Land, two continuous metrics, relative bias (RB) and Kling-Gupta efficiency (KGEʹ), were used. RB describes the deviation of IMERG and ERA5-Land from gauge data. RB range is from − ∞ to ∞ and perfect value is 0. The sign of value (positive or negative) of RB represents the direction of deviation (overestimation or underestimation) of IMERG and ERA5-Land compared with gauge precipitation. The magnitude of the absolute value of RB means magnitude of deviation (magnitude of overestimation or underestimation). KGEʹ^54,55^ describes overall accuracy, which is a multi-component metric integrating the correlation (CC), bias ratio ($\beta$), and variability ratio ($\gamma$), proposed by Gupta et al. (2009) and revised by Kling et al. (2012). The range and perfect value of KGEʹ are (− ∞, 1] and 1, respectively. The higher value of KGEʹ, the better accuracy.

1 $$RB=\frac{\sum_{i=1}^{N}({Y}_{i}-{X}_{i})}{{\sum }_{i=1}^{N}{X}_{i}}\times 100,$$

2 $$KG{E}^{^{\prime}}=1-\sqrt{{\left(CC-1\right)}^{2}+{\left(\beta -1\right)}^{2}+{\left(\gamma -1\right)}^{2}},$$

where *N* represents the number of samples; ${X}_{i}$ and ${Y}_{i}$ represent the *i*th gauge record and IMERG/ERA5-Land estimate, respectively; ${\mu }_{X}$ and ${\mu }_{Y}$ are the corresponding mean values; ${\sigma }_{X}$ and ${\sigma }_{Y}$ are the corresponding standard deviations; $CC=\sum_{i=1}^{N}({X}_{i}-{\mu }_{X})({Y}_{i}-{\mu }_{Y})/\sqrt{{\sum }_{i=1}^{N}{({X}_{i}-{\mu }_{X})}^{2}}\sqrt{{\sum }_{i=1}^{N}{({Y}_{i}-{\mu }_{Y})}^{2}}$; $\beta ={\mu }_{Y}/{\mu }_{X}$; $\gamma =({\mu }_{Y}/{\sigma }_{Y})/({\mu }_{X}/{\sigma }_{X})$.

Referencing China’s national standard on the grade of precipitation (GB/T 28592-2012 which can be consulted at http://openstd.samr.gov.cn/), daily precipitation can be divided into 5 classes (Table 2): no precipitation, light precipitation, moderate precipitation, heavy precipitation, and extreme precipitation. Two categorical metrics (frequency bias (FB) and critical success index (CSI)) are used to further quantify the performance detection ability of IMERG and ERA5-Land for the occurrence of precipitation events falling in different precipitation classes. FB shows the extent to which IMERG and ERA5-Land overestimate or underestimate the amount of precipitation events. The range and perfect value of FB are [− 1, ∞) and 0, respectively. Similar to RB, the sign of value and the magnitude of the absolute value of FB provide information on the direction of deviation and magnitude of deviation of IMERG and ERA5-Land in the amount of precipitation events. CSI combines the features of the probability of detection (POD) and the false alarm ratio (FAR) to demonstrate the overall detection ability of target datasets. The range and perfect value of CSI are [0,1] and 1, respectively. The closer of CSI to 1, the better detectability.

3 $$FB=\frac{HIT+FALSE}{HIT+MISS}-1,$$

4 $$CSI=\frac{1}{1/\left(1-FAR\right)+1/POD-1}=\frac{HIT}{HIT+MISS+FALSE},$$

where $HIT$ is the frequency of events that are detected and observed; $MISS$ is the frequency of events that are observed but not detected; $FALSE$ is the frequency of events that are detected but not observed; $POD=HIT/(HIT+MISS)$; $FAR=FALSE/(HIT+FALSE)$.

**Table 2:** **C**lassification of precipitation based on daily bias-corrected gauge data.

Precipitation class	Daily precipitation (mm/day)
No precipitation	< 0.1
Light precipitation	0.1–10
Moderate precipitation	10–25
Heavy precipitation	25–50
Extreme precipitation	> 50

#### Precipitation phase classification method

The bias of gauge measurements is related to the precipitation phase. In general, the bias for rainfall is relatively small and snowfall is greatly affected by wind speed^56^. Therefore, before correcting the bias of gauge measurements, the precipitation phase should be determined first. There are two types of precipitation phase classification schemes^57^. The first type only uses air temperature as the basis to classify precipitation phases^32,36,37,58^. This type of schemes requires relatively few parameters and is suitable for data-scarce areas. However, the temperature thresholds used to classify precipitation phases can vary significantly in different areas. Unlike the first type, the second type also considers other parameters such as wet-bulb temperature and relative humidity in addition to air temperature^59–61^. Sims and Liu^61^ found that the wet-bulb temperature, rather than ambient air temperature, should be used to separate solid and liquid precipitation. This is because the wet-bulb temperature is closer to the actual temperature of precipitation particles, and it has a smaller range for which there is uncertainty as to whether the precipitation is solid or liquid. Based on the dependence of the precipitation phase on different geophysical parameters, they developed a parameterization scheme over a global scale that accepts 2-m temperature, relative humidity and surface pressure (to calculate wet-bulb temperature), low-level vertical temperature lapse rate, surface skin temperature, and surface type as inputs and returns the conditional probability of solid precipitation. However, the required parameters for this scheme are too many and cannot be obtained from weather stations alone. For example, the calculation of vertical temperature lapse rate needs observations from radiosondes and balloons. Ding et al.^60^ investigated the relationship of precipitation phases with surface elevation and meteorological variables. Based on the results, a parameterization scheme was developed to discriminate the precipitation phase, with input of daily mean wet-bulb temperature, relative humidity, and surface elevation. They also compared the performance of this scheme with those of 11 other schemes for China territory and the evaluations showed that the new scheme gives better accuracy with respect to other schemes. Moreover, the number of required parameters for this scheme is moderate and all parameters can be provided by weather stations. Therefore, the precipitation phase classification in this paper was based on this scheme. This scheme uses the wet-bulb temperature instead of air temperature to classify the precipitation phase. Two threshold wet-bulb temperatures are calculated with inputs of relative humidity and surface elevation. Under different values of relative humidity and surface elevation, the thresholds also vary. In other words, this parameterization scheme is a dynamic threshold method. The details are as follows.

Two threshold wet-bulb temperatures (${T}_{min}$ and ${T}_{max}$) are defined such that the precipitation phase is decided by:

5 $$\text{Phase}=\left\{\begin{array}{l}{\text{snowfall}}, \quad \text{if } {T}_{w}\le {T}_{min}\\ {\text{mixed}}, \quad \text{if }\, {T}_{min}<{T}_{w}<{T}_{max}\\ {\text{rainfall}} \quad {, \text{if }T}_{w}\ge {T}_{max}\end{array}\right.,$$

where ${T}_{w}$ (℃) is the wet-bulb temperature.

${T}_{w}$ is calculated by:

6 $${T}_{w}={T}_{d}-\frac{{e}_{sat}\left({T}_{d}\right)\left(1-RH\right)}{0.000643{p}_{s}+\frac{\partial {e}_{sat}}{\partial {T}_{d}}},$$

where ${T}_{d}$ is daily mean air temperature (℃); $RH$ is relative humidity and it ranges from 0 to 1;${p}_{s}$ is air pressure (hPa); ${e}_{sat}\left({T}_{d}\right)$ is the saturated vapor pressure (hPa) at ${T}_{d}$ and is given by Tetens’s empirical formula^62^ as:

7 $${e}_{sat}\left({T}_{d}\right)=6.1078\text{exp}\left(\frac{17.27{T}_{d}}{{T}_{d}+237.3}\right),$$

The calculation of ${T}_{min}$ and ${T}_{max}$ is shown in the following formulas:

8 $$ T_{{min}}  = \left\{ {\begin{array}{*{20}l}    {T_{0}  - \Delta S \times \ln \left[ {\exp \left( {\frac{{\Delta T}}{{\Delta S}}} \right) - 2\exp \left( { - \frac{{\Delta T}}{{\Delta S}}} \right)} \right]} \hfill & {\frac{{\Delta T}}{{\Delta S}} > \ln 2} \hfill  \\    {T_{0} } \hfill & {\frac{{\Delta T}}{{\Delta S}} \le \ln 2} \hfill  \\   \end{array} } \right., $$

9 $$ T_{{max}}  = \left\{ {\begin{array}{*{20}l}    {2T_{0}  - T_{{min}} } & {\frac{{\Delta T}}{{\Delta S}} > \ln 2}  \\    {T_{0} } & {\frac{{\Delta T}}{{\Delta S}} \le \ln 2}  \\   \end{array} } \right., $$

where ${T}_{0}$, $\Delta T$, and $\Delta S$ are given by:

10 $$\Delta T=0.125-0.099RH+1.018{RH}^{2},$$

11 $$\Delta S=2.374-1.634RH,$$

12 $${T}_{0}=-\,5.87-0.1042Z+0.0885{Z}^{2}+16.06RH-9.614{RH}^{2},$$

where $Z$ denotes station elevation (km).

#### Bias-correction method

Due to the negative bias inherent in gauge data, the reliability of the performance evaluation of IMERG and ERA5-Land directly using gauge data as the benchmark will also be weakened. Fortunately, there have been a lot of research works for the bias-correction methods of gauge data^32,34,36–38^.

According to Sevruk and Hamon^63^, bias correction for gauge precipitation mainly includes trace precipitation, wetting losses, evaporation losses, and wind undercatch errors. The general formula for precipitation bias correction is written as follows^28,36,63^:

13 $${P}_{c}=K\left({P}_{g}+{\Delta P}_{w}+{\Delta P}_{e}+{\Delta P}_{t}\right),$$

where ${P}_{c}$ is the corrected gauge precipitation; ${P}_{g}$ is the measured gauge precipitation; ${\Delta P}_{w}$, ${\Delta P}_{e}$, and ${\Delta P}_{t}$ are wetting losses, evaporation losses, and trace precipitation, respectively; and $K$ is the correction coefficient for wind-induced errors, which is defined as 1/CR, and CR is the catch ratio due to wind-induced undercatch.

Evaporation losses are strongly dependent on weather conditions and methods of observations^64^. Because daily variation and seasonal change in evaporation losses are great and can be very site-dependent, it is difficult to give an incredible estimate of the daily evaporation losses at regional station networks by using the averaging evaporation amount obtained from other experiment sites. In addition, the annual evaporation loss in China is expected to be small due to the use of a funnel and a container in the high-evaporation period^36^. Therefore, we neglected this error in this study.

A precipitation event with < 0.10 mm is beyond the resolution of CSPG and is recorded as trace amount of precipitation. Trace events are counted as precipitation days but treated as zero amounts. Sometimes, two trace precipitation events are reported in a single precipitation day. Considering the wetting loss, the gauge cannot detect the precipitation events that are less than the wetting loss. To be conservative, we didn’t correct the wetting loss and wind undercatch for trace precipitation days. Following Ye et al.^36^ and Zhang et al.^37^, a value of 0.10 mm was assigned for any given trace day, regardless of the number of trace observations reported. In the gauge records, trace precipitation is flagged as 32,700.

Wetting losses depend on gauge types, precipitation phases, the number of observations, and the gauge-emptied times. The wetting loss experiments in the Urumqi river basin show the average wetting loss of CSPG per observation was 0.23 mm, 0.29 mm, 0.30 mm for rainfall, mixed precipitation, and snowfall, respectively^38,65^. To be conservative, we corrected wetting losses once a precipitation day regardless of the number of observations in a day. These correction amounts for wetting losses were also applied in the works of Ye et al.^36^ and Zhang et al.^37^. The correction for wetting losses was trigged for measurable precipitation days (gauge-measured precipitation ≥ 0.10 mm).

The wind-induced errors are caused by the wind field deformation over the gauge orifice^63^. It has been well documented that the wind undercatch for snowfall is much higher than for rainfall under the same wind speed^36,66^. It is essential to classify the phase of precipitation in order to apply an appropriate wind-loss correction for different precipitation phases. In this work, information on the precipitation phase was not available in the station metadata. Alternatively, we employed the parameterization scheme developed by Ding et al.^60^ to discriminate the precipitation phase. The details of this scheme have been presented in “Precipitation phase classification method” section. Catch ratio was defined as the ratio of the amount of precipitation caught by a gauge (including the record amount and wetting loss) to the true precipitation. The catch ratio equation we applied is based on Yang et al.^38^ who conducted gauge intercomparison experiments in the Urumqi river basin. The octagonal vertical double fence (DFIR) was used as reference measurements^38,57^. According to the intercomparison study, the catch ratios (percent) for different precipitation phases as the function of daily mean wind speed at 10 m height were established.

14 $${CR}_{snow}=100\text{exp}\left(-\,0.056{U}_{10}\right),$$

15 $${CR}_{rain}=100\text{exp}\left(-\,0.041{U}_{10}\right),$$

where ${CR}_{snow}$ and ${CR}_{rain}$ are catch ratios for snowfall, rainfall, and mixed precipitation, respectively; ${U}_{10}$ is the daily mean wind speed at 10 m height.

For mixed precipitation, the catch ratio was estimated by a linear relationship of the catch ratios for snowfall and rainfall determined by daily mean temperature (${T}_{d}$)^36^:

16 $${CR}_{mixed}={CR}_{snow}-({CR}_{snow}-{CR}_{rain})\times ({T}_{d}+2)/4.$$

The correction for wind-induced errors was also trigged for measurable precipitation days.

Finally, the corrected precipitation can be obtained through the following formula:

17 $$ P_{c} = \left\{ {\begin{array}{*{20}l} {\left( {P_{m} + \Delta P_{w} } \right)/CR} \hfill & {{\text{for measurable day }}(P_{m} \ge 0.1\;{\text{mm}})} \hfill \\ {\Delta P_{t} } \hfill & {{\text{for trace precipitation day}}\;\left( {0 < P_{m} < 0.1\;{\text{mm}}} \right)} \hfill \\ \end{array} } \right.. $$

The procedure of bias-correction method can be summarized as follows:

1. *Data collection* Twelve years (2005–2016) of the daily gauge precipitation data from CSPG installed in 814 weather stations operated by CMA were obtained. In addition, the daily mean air temperature, relative humidity, air pressure, station elevation, and daily mean wind speed at 10 m height from these CMA stations were also gathered.
2. *Precipitation phase classification* The daily mean air temperature, relative humidity, air pressure, and station elevation were used to classify precipitation phase according to Eqs. (5)–(12)
3. *Calculating each bias correction component* Evaporation loss was neglected. For each measurable precipitation day, wetting losses and wind-induced errors were corrected. The correction amounts of wetting losses were 0.23 mm, 0.29 mm, and 0.30 mm for rainfall, mixed precipitation, and snowfall, respectively. The catch ratios for snowfall, rainfall, and mixed precipitation were calculated using daily mean speed at 10 m height and daily mean temperature according to Eqs. (14)–(16), respectively. For trace precipitation days, only correction for trace precipitation was made and a value of 0.1 mm was assigned.
4. *Removing bias in gauge data* After calculating each bias correction component, bias-corrected gauge data can be produced according to Eq. (17).

### Consent to publish

All the authors have approved the submission and consented for the publication.

## Results

### Spatial distribution of annual mean precipitation

The spatial distribution of annual mean precipitation from IMERG, ERA5-Land, Gauge_O, and Gauge_C over China is shown in Fig. 2. All four precipitation datasets (IMERG, ERA5-Land, Gauge_O, and Gauge_C) display a southeast-to-northwest decreasing trend over China. This result demonstrates that both IMERG and ERA5-Land can capture spatial trend of precipitation over China and negative bias in gauge data doesn’t influence spatial trend of gauge precipitation. However, comparing Gauge_O with Gauge_C, we found that the precipitation belts with lower annual mean precipitation slightly shift in the direction of the precipitation belts with higher annual mean precipitation after bias correction of gauge data. For example, the precipitation belt with precipitation ranging from 250 to 500 mm/year is replaced by precipitation belt with precipitation ranging from 500 to 1000 mm/year in the border of these two precipitation belts. This slightly shifting of precipitation belts results from the increasing precipitation from gauge data after removing negative bias. After bias correction, annual mean precipitation from gauge increases everywhere. Under the same standard for precipitation belt classification, the precipitation belt of one place might be replaced by the precipitation belt with higher annual mean precipitation.

### The correction for bias in gauge measurements

We calculated the correction amount (CA) and correction factor (CF) in different locations, climatic zones, seasons, and precipitation phases. The results are displayed in Fig. 3. The correction amount was calculated as $\text{Gauge}\_\text{C}-\text{Gauge}\_\text{O}$. The correction factor was calculated as $(\text{Gauge}\_\text{C}-\text{Gauge}\_\text{O})/\text{Gauge}\_\text{O}$.

The spatial trend of correction amount (Fig. 3a) matches that of Gauge_O (Fig. 2c), with large correction amount in southeastern China where annual precipitation is high and small correction amount in northwestern China where annual precipitation is low. The correction amount and Gauge_O both gradually decrease from severe-humid to severe-arid climatic zone (Fig. 3c). On seasonal scale, the correction amount and largest Gauge_O both have largest values in summer and smallest values in winter (Fig. 3e). For different precipitation phases, the values of the correction amount and Gauge_O in descending order both occur in rainfall, mixed precipitation, and snowfall (Fig. 3g). These results demonstrate that there is some kind of positive correlation between the correction amount and gauge-observed precipitation. The reason is probably that the corrected gauge precipitation is proportional to the measured gauge precipitation (Eq. (13)).

As for the correction factor, the situation is different. The spatial trend of the correction factor (Fig. 3b) is opposite to that of the correction amount (Fig. 3a), with large correction factor in northwestern China and small correction factor in southeastern China. From severe-humid to severe-arid climatic zone, correction factor increases from 11.3 to 25.6% (Fig. 3d). On seasonal scale, the correction factor has largest value in winter and smallest value in summer (Fig. 3f). Among three precipitation phases, the value of the correction factor from largest to smallest occurs in snowfall, mixed precipitation, and rainfall. The snowfall proportion in precipitation events and the lower catch efficiency of snowfall than rainfall make some contribution to these results. The snowfall proportion decreases from northwestern China (arid climates) to southeastern China (humid climates). Rainfall is dominant in summer and most snowfall occurs in winter. The catch ratio of snowfall is lower than rainfall under the same wind speed. Moreover, the correction factor is 12.7% on average in China, which is no more than those in the works of Li et al.^32^ (17.9%), Yao et al.^41^ (17.7%), and Ye et al.^36^ (19%). The difference can be explained by the different station numbers and data periods. The station numbers (data periods) in Li et al.^32^, Yao et al.^41^, and Ye et al.^36^ are 553 (1961–2015), 552 (1961–2015), and 710 (1951–1998), respectively, compared to 814 (2005–2016) in this study.

### Spatial variation of continuous evaluation metrics

We calculated continuous metrics before bias correction and after bias correction by using Gauge_O and Gauge_C as the benchmark, respectively. RBs before bias correction and after bias correction are denoted by RB_O and RB_C. KGEʹ_O or KGEʹ_C is for KGEʹ likewise. We also calculated the difference between continuous metrics after bias correction and those before bias correction. For RB, the difference was labeled as ∆RB (RB_C minus RB_O). For KGEʹ, the difference was labeled as ∆KGEʹ (KGEʹ_C minus KGEʹ_O).

RB_O, RB_C, and ∆RB for IMERG and ERA5-Land were calculated at each weather station and their distributions were presented in Fig. 4. The spatial distributions of RB_O and RB_C for IMERG (Fig. 4a,b) both indicate that IMERG underestimates precipitation in southern China and Qinghai-Tibet Plateau while overestimates precipitation in northern China. Moreover, the spatial distributions of RB_O and RB_C for ERA5-Land (Fig. 4d,e) both show that the precipitation of ERA5-Land is overestimated in Tibet Plateau and Yunnan-Guizhou Plateau.

However, comparing the spatial distribution of ∆RB for IMERG (Fig. 4c) and ERA5-Land (Fig. 4f), it is shown the noticeable decline of RB for IMERG and ERA5-Land occurs everywhere and the magnitude of decline varies spatially after bias correction. The magnitude of decline of RB for IMERG is greater in northern China than in southern China while that for ERA5-Land is greater in western China than in eastern China. The reason for the obvious decline of RB is that the benchmark dataset, the precipitation from gauge data, increases after bias correction. This decline of RB has led to the sign of RB reverses and the absolute value of RB changes, that is, the direction of deviation and magnitude of deviation change. For example, in comparison with Fig. 4a,b, many medium red (20% < RB < 50%) and heavy red points (RB > 50%) are replaced by light red (0 < RB < 20%) and medium red points, respectively, in northeastern China after bias correction. This indicates the magnitude of overestimation of IMERG precipitation reduces in northeastern China. In the comparison with Fig. 4d,e, many light red points are replaced by light blue points (− 20% < RB < 0) in eastern China after bias correction. The result demonstrates that the direction of deviation of ERA5-Land precipitation changes from overestimation to underestimation in eastern China.

Figure 5 shows the spatial distributions of KGEʹ_O, KGEʹ_C, and ∆KGEʹ for IMERG and ERA5-Land, respectively. As can be seen from Fig. 5c–f, it can be found: After bias correction, that the accuracy of IMERG deteriorates in southern China and Tibetan Plateau with ∆KGEʹ < 0 but improves in the remaining regions of China with ∆KGEʹ > 0. ERA5-Land performs better in most of China except the eastern coast of China and isolated sites inland. However, the spatial trends of KGEʹ for IMERG and ERA5-Land are almost unaffected by systematic bias in gauge data. Comparing the spatial distribution of KGEʹ_O and KGEʹ_C for IMERG (Fig. 5a,b) and for ERA5-Land (Fig. 5d,e), respectively, we found the accuracy of IMERG shows the southeast-to-northwest-decreasing trend and that of ERA5-Land shows the northeast-to-southwest-decreasing trend whether before or after bias correction. There are two reasons for the above two distinct spatial trends of accuracy. First, satellites (reanalysis) exhibit superior performance at low (high) latitudes dominated by intense, localized convective (persistent, large-scale stratiform) precipitation system^67–71^. This explains the south-to-north-decreasing (north-to-south-decreasing) trend of accuracy for IMERG (ERA5-Land). Another reason is the increasingly sophisticated underlying surfaces from the eastern coast to the western inland which explains the east-to-west-decreasing trend of accuracy for two precipitation estimates.

### Inter-annual and seasonal variation of continuous metrics

**Figure 6:**
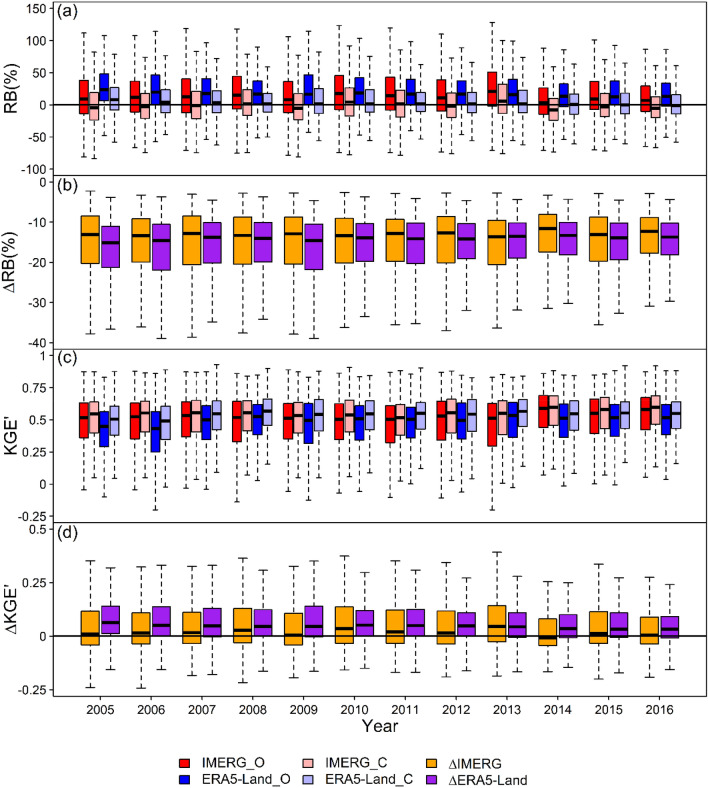
Boxplots of (**a**) RB (RB_O and RB_C), (**b**) ∆RB, (**c**) KGEʹ (KGEʹ_O and KGEʹ_C), and (**d**) ∆KGEʹ for IMERG and ERA5-Land at inter-annual scale.

**Figure 7:**
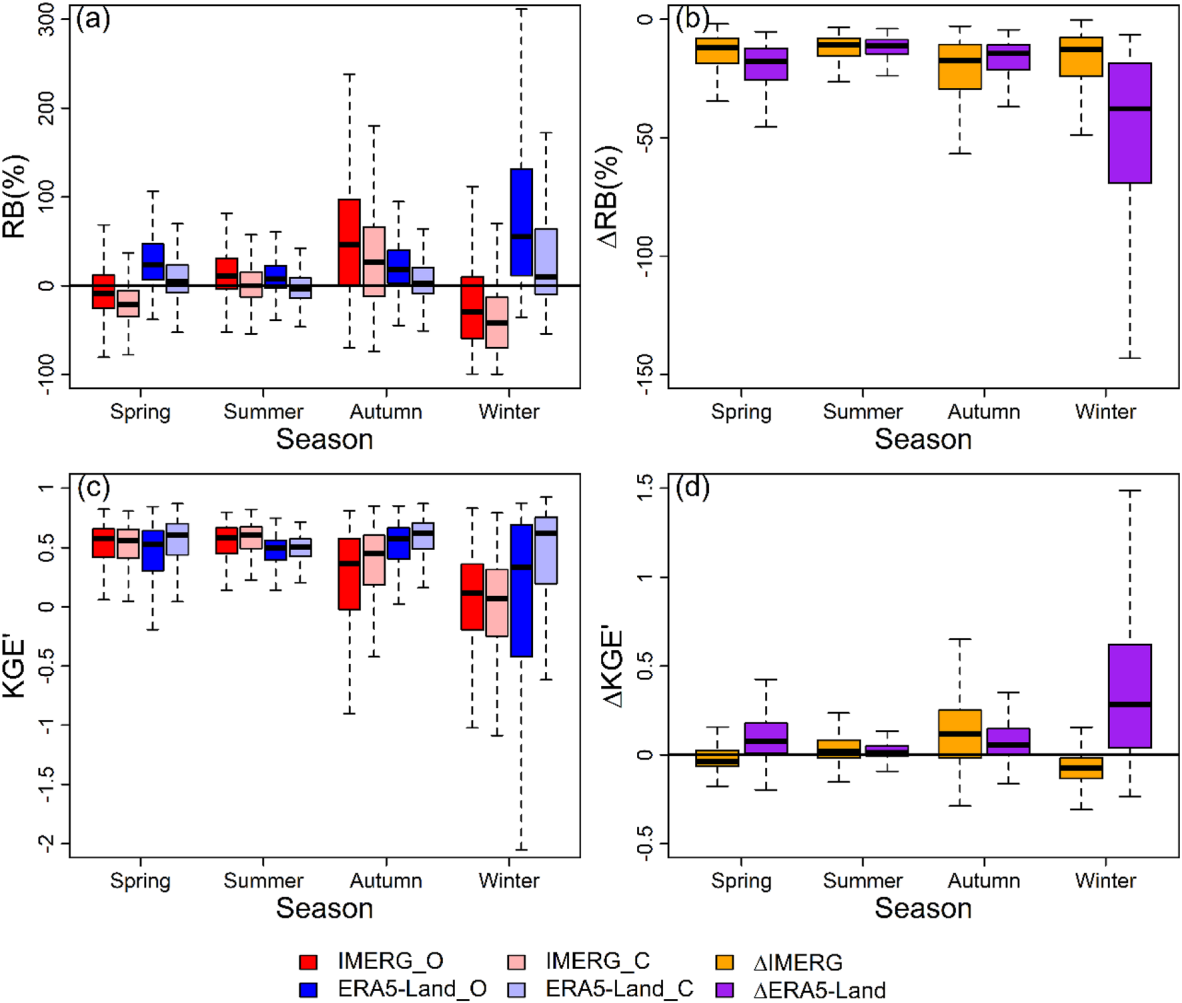
Boxplots of (**a**) RB (RB_O and RB_C), (**b**) ∆RB, (**c**) KGEʹ (KGEʹ_O and KGEʹ_C), and (**d**) ∆KGEʹ for IMERG and ERA5-Land at the seasonal scale.

We calculated inter-annual and seasonal RB_O, RB_C, KGEʹ_O, KGEʹ_C, ∆RB, and ∆KGEʹ for IMERG and ERA5-Land at each weather station. The boxplots of them are plotted in Figs. 6 and 7. IMERG_O and ERA5-Land_O stand for continuous metrics (RB_O and KGEʹ_O) calculated by using Gauge_O for IMERG and ERA5-Land, respectively. IMERG_C and ERA5-Land_C stand for continuous metrics (RB_C and KGEʹ_C) calculated by using Gauge_C for IMERG and ERA5-Land, respectively. ∆IMERG and ∆ERA5-Land stand for the difference between continuous metrics (∆RB and ∆KGEʹ) after bias correction and those before bias correction for IMERG and ERA5-Land, respectively. We defined March–May, June–August, September–November, and December–February as spring, summer, autumn, and winter.

As can be seen from Figs. 6a and 7a, the RBs for IMERG and ERA5-Land both decline noticeably in all years and all seasons after bias correction with the upper staple of each box in Figs. 6b and 7b below ∆RB = 0. The medians of ∆RB for IMERG and ERA5-Land range from − 13.7 to − 11.6% and from − 15.2 to − 13.3%, respectively, between 2005 and 2016. At seasonal scale, the medians of ∆RB for IMERG and ERA5-Land range from − 17.4 to − 10.7% and from − 37.8 to − 11.1%, respectively. These declines have resulted in the change of magnitude of deviation and the reverse of direction of deviation in inter-annual and seasonal scales. In the inter-annual scale, the signs of median RBs for IMERG change from positive to negative in 2005, 2009, 2014, and 2016. The absolute values of median RB for ERA5-Land reduce every year. On the seasonal scale, the absolute values of median RB for IMERG increase in spring and winter but reduce in summer and autumn. The absolute values of median RB for ERA5-Land reduce in all four seasons. Obviously, the RB decline will inevitably lead to the change of deviation degree. The reverse of deviation state depends on the magnitude of RB decline. If RB_O is greater than 0 and the magnitude of RB decline is greater than the absolute value of RB_O, the reverse of direction of deviation will occur.

From Fig. 6c,d, the overall accuracies for IMERG and ERA5-Land both slightly increase in all years and the magnitude of improvement for ERA5-Land is a little larger than IMERG. The medians of ∆KGEʹ for IMERG and ERA5-Land range from 0 to 0.05 and from 0.03 to 0.06, respectively, and the median line of ∆KGEʹ for ERA5-Land is higher than that for IMERG for each year between 2005 and 2016. From Fig. 7c,d, IMERG performs worse in spring and winter with the median ∆KGEʹ =  − 0.04 and − 0.08, respectively, but performs better in summer and autumn with the median ∆KGEʹ = 0.02 and 0.12, respectively. For ERA5-Land, the overall accuracy increases in all four seasons with ∆KGEʹ ranging from 0.01 to 0.28. In addition, comparing KGEʹ_O with KGEʹ_C for IMERG and ERA5-Land in Fig. 7c, it can be concluded that ERA5-Land has better overall accuracy than IMERG in autumn and winter while the contrary is the case in summer whether before bias correction or after bias correction. In spring, the performance superiority between IMERG and ERA5-Land reverses after bias correction. Before bias correction, the median KGEʹ of IMERG (0.58) is greater than that of ERA5-Land (0.53). After bias correction, the median KGEʹ of IMERG (0.56) becomes less than that of ERA5-Land (0.61).

### Accuracy of IMERG and ERA5-Land over different climatic zones

To explore the regional influence of negative bias in gauge data on the accuracy evaluation of IMERG and ERA5-Land, we computed overall RB_O, RB_C, ∆RB, KGEʹ_O, KGEʹ_C, and ∆KGEʹ for IMERG and ERA5-Land over the whole China (Table 3) and different climatic zones (Table 4).

**Table 3:** Overall continuous metrics for IMERG and ERA5-Land over the whole of China.

Product	RB			KGEʹ		
	RB_O (%)	RB_C (%)	∆RB (%)	KGEʹ_O	KGEʹ_C	∆KGEʹ
IMERG	6.8	− 5.2	− 12.0	0.69	0.69	0
ERA5-Land	15.0	2.1	− 12.9	0.52	0.56	0.04

**Table 4:** Overall continuous metrics for IMERG and ERA5-Land over the different climatic zones.

Climatic zone	Product	RB			KGEʹ		
		RB_O (%)	RB_C (%)	∆RB (%)	KGEʹ_O	KGEʹ_C	∆KGEʹ
Severe-humid	IMERG	− 5.1	− 14.8	− 9.7	0.70	0.66	− 0.04
	ERA5-Land	− 0.2	− 10.4	− 10.2	0.56	0.54	− 0.02
Humid	IMERG	5.9	− 4.8	− 10.7	0.70	0.70	0
	ERA5-Land	18.6	6.6	− 12.0	0.49	0.53	0.04
Semi-humid	IMERG	13.1	− 0.1	− 13.0	0.63	0.65	0.02
	ERA5-Land	28.8	13.7	− 15.1	0.46	0.54	0.08
Semi-arid	IMERG	22.5	5.9	− 16.6	0.58	0.64	0.06
	ERA5-Land	26.7	9.5	− 17.2	0.49	0.58	0.09
Arid	IMERG	12.8	− 6.8	− 19.6	0.57	0.59	0.02
	ERA5-Land	39.2	15.0	− 24.2	0.40	0.56	0.16
Severe-arid	IMERG	88.9	50.4	− 38.5	− 0.03	0.29	0.32
	ERA5-Land	50.7	20.0	− 30.7	0.29	0.51	0.22

As can be seen from Table 3, over the whole of China, the overall RBs of IMERG and ERA5-Land both decline after bias correction. The direction of deviation of IMERG changes from overestimation (RB_O = 6.8%) to underestimation (RB_C =  − 5.2%), and the magnitude of deviation of ERA5-Land remarkably reduces, changing from significant overestimation to slight overestimation. However, the accuracies of IMERG and ERA5-Land generally do not change much.

As shown in Table 4, after bias correction, the overall RBs of IMERG and ERA5-Land again decline over different climatic zones and the magnitude of RB decline gradually rise from severe-humid climatic zone to severe-arid climatic zone along with ∆RB for IMERG varying from − 9.7 to − 38.5% and ∆RB for ERA5-Land varying from − 10.2 to − 30.7%. In other words, the RB declines more in arid climates than in humid climates. This result probably attributes to more trace precipitation events, lower catch ratio for wind-induced undercatch, and more correction percentage for wetting loss which are contributed by scarce precipitation, higher wind speed, and a greater proportion of snowfall events, respectively, in arid climates with respect to humid climates over China^37^. Moreover, the changes of direction of deviation and magnitude of deviation for IMERG and ERA5-Land also occur. For example, the magnitude of deviation of IMERG changes from slight underestimation (RB_O =  − 5.1%) to significantly underestimation (RB_C =  − 14.8%) over severe-humid climate zone and the direction of deviation of IMERG changes from overestimation to underestimation over the humid climatic zone.

Over the severe-humid zone, the overall accuracies of IMERG and ERA5-Land both slightly deteriorate after bias correction with ∆KGEʹ =  − 0.04 and ∆KGEʹ =  − 0.02, respectively. Over the remaining five climatic zones, the overall accuracies of IMERG and ERA5-Land all improve, especially in the severe-arid climatic zone for IMERG with ∆KGEʹ = 0.32 and in arid and severe-arid climatic zone for ERA5-Land with ∆KGEʹ = 0.16 and 0.22. It should be noted the overall accuracies of IMERG in severe-arid climatic zone is poor, probably due to sub-cloud evaporation^72^. Over arid regions, the atmosphere beneath the clouds is mostly dry. As a result, precipitation detected by satellite will evaporate before reaching the surface resulting in huge overestimation of surface precipitation. This phenomenon is severe in the severe-arid climatic zone with RB_O = 88.9% and RB_C = 50.4%.

### Detection ability of IMERG and ERA5-Land in different classes of precipitation events

**Figure 8:**
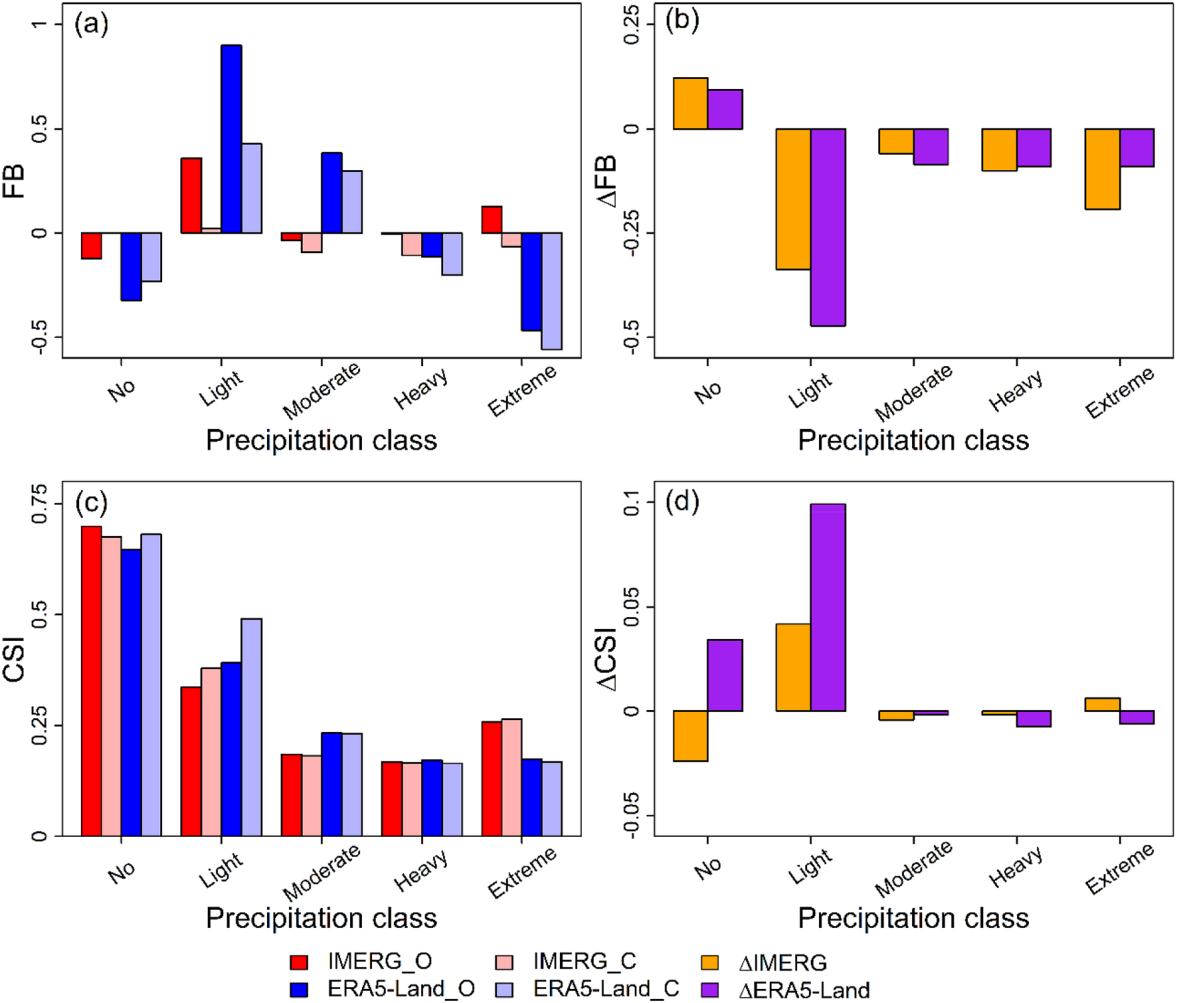
Bar chart of (**a**) FB (FB_O and FB_C), (**b**) ∆FB, (**c**) CSI (CSI_O and CSI_C), and (**d**) ∆CSI for IMERG and ERA5-Land in different precipitation classes.

To investigate the influence of systematic error in gauge data on the frequency distribution of gauge-observed precipitation events and categorical metrics for IMERG and ERA5-Land, we listed the frequency distributions of precipitation events in different precipitation classes from Gauge_O and Gauge_C in Table 5 and calculated FB_O, FB_C, ∆FB, CSI_O, CSI_C, and ∆CSI for IMERG and ERA5-Land in different precipitation classes which are shown in Fig. 8. Similar to RB_O, RB_C, ∆RB, KGEʹ_O, KGEʹ_C, and ∆KGEʹ, FB_O and CSI_O denote categorical metrics calculated before bias correction, FB_C and CSI_C denote categorical metrics calculated after bias correction, and ∆FB and ∆CSI denote the change of categorical metrics after bias correction. Analogously, IMERG_O, ERA5-Land_O, IMERG_C, ERA5-Land_C, ∆IMERG, and ∆ERA5-Land in Fig. 8 have similar meanings to those in Figs. 6 and 7 except that they stand for categorical metrics.

**Table 5:** Frequency distributions of precipitation events in different precipitation classes from Gauge_O and Gauge_C.

Gauge precipitation	No	Light	Moderate	Heavy	Extreme	Total
Gauge_O	2,480,129	844,130	155,678	60,226	23,667	3,563,830
Gauge_C	2,179,141	1,123,306	165,854	66,994	28,535	3,563,830
∆Gauge	− 300,988	279,176	10,176	6768	4868	0

∆Gauge denotes the frequency change for gauge-observed precipitation events after bias correction, which is calculated by frequency distribution from Gauge_C minus that from Gauge_O.

As can be seen from Table 5, after bias correction, the apparent change of the frequency distribution of gauge-observed precipitation events in different precipitation classes occurs. In no precipitation class, the amount of precipitation events remarkably decreases. In the remaining five precipitation classes, the amounts of precipitation events both increase, especially in the light precipitation class. The obvious changes of precipitation events in no and tiny precipitation classes might be mainly caused by the change of the precipitation class of trace precipitation events. Before bias correction, trace precipitation events considered as zero precipitation are classified into no precipitation class. After bias correction, the precipitation intensity of trace precipitation events is corrected to 0.1 mm and thus trace precipitation events are classified into light precipitation events.

The change in the frequency distribution of gauge-observed precipitation events inevitably affects categorical metrics for IMERG and ERA5-Land. From Fig. 8a,b, the FBs for IMERG and ERA5-Land increase in no precipitation class but decrease in the other four precipitation classes after bias correction. This change is in accordance with the change in the frequency distribution of gauge-observed precipitation events. As a result, the magnitude of deviation and direction of deviation in the amount of the different classes of precipitation events also alter. For example, the magnitudes of overestimation both reduce for IMERG and ERA5-Land in the amount of light precipitation events, with FB_O = 0.36 and FB_C = 0.02 for IMERG and FB_O = 0.90 and FB_C = 0.43 for ERA5-Land. In the amount of extreme precipitation events, the direction of deviation for IMERG changes from overestimation to underestimation, with FB_O = 0.13 and FB_C =  − 0.06.

From Fig. 8c,d, the CSIs for IMERG and ERA5-Land nearly have no change in moderate, heavy, and extreme precipitation classes with |∆CSI|≤ 0.008 which indicates negative bias in gauge data almost has little influence on the evaluation of detection ability for IMERG and ERA5-Land in these precipitation classes. In other words, IMERG has better capability in detecting moderate precipitation events, ERA5-Land has better performance in detecting extreme precipitation events, and both two products have the comparable ability in detecting heavy precipitation events. It should be noted that the performance of IMERG in detecting extreme events is better than ERA5-Land and their performances in detecting heavy events are comparable. This demonstrates that satellite precipitation products maybe more suitable than reanalysis precipitation products for flood forecast and warning due to the detection of heavy and extreme precipitation vital for flood-related applications^73^.

Whereas, the noticeable changes of CSI in no and light precipitation events have taken place after bias correction. The CSI of IMERG decreases but that of ERA5-Land increases in no precipitation events which weakens the superiority of detection ability of IMERG over that of ERA5-Land in the class of no precipitation events. Before bias correction, IMERG has an advantage over ERA5-Land in detecting no precipitation events, with CSI_O = 0.70 for IMERG and CSI_O = 0.65 for ERA5-Land. After bias correction, the detection ability of IMERG in no precipitation events is comparable to that of ERA5-Land, with CSI_O = 0.68 both for IMERG ERA5-Land. In light precipitation events, the CSIs of two products both increase and the increasing magnitude of ERA5-Land is more significant than that of IMERG with ∆CSI = 0.04 for IMERG and ∆CSI = 0.10 for ERA5-Land.

### Accuracy of IMERG and ERA5-Land for different phases of precipitation

Last, we also assess the performance accuracy of IMERG and ERA5-Land for different precipitation phases by calculating the corresponding continuous metrics. The results are listed in Table 6. After bias correction, the RBs for rainfall, mixed precipitation, and snowfall all decrease. The magnitude of RB decline for snowfall is much greater than that for rainfall. This might result from the different wind-induced undercatch for different precipitation phases. First, under the same wind speed, gauge wind-induced undercatch of snowfall is much higher than rainfall^35,36,74^, which is reflected by the catch ratio calculating equations (Eqs. (14)–(16)). Second, in China, the high wind speed area^75^ coincides with major snowfall area^20^ (Xinjiang, Tibetan Plateau, and northeastern China). Thus, snowfall is usually accomplished by higher wind speed and higher wind-induced undercatch compared with rainfall. Again, the RB decline has resulted in the change of the direction and magnitude of deviation of IMERG and ERA5-Land for different precipitation phases. For instance, the direction of deviation of IMERG changes from overestimation to underestimation and the magnitude of deviation of ERA5-Land changes from apparent overestimation to slight underestimation for rainfall.

**Table 6:** Continuous metrics of IMERG and ERA5-Land for different precipitation phases.

Precipitation phase		RB			KGEʹ		
		RB_O (%)	RB_C (%)	∆RB (%)	KGEʹ_O	KGEʹ_C	∆KGEʹ
Rainfall	IMERG	7.9	− 3.7	− 11.6	0.68	0.69	0.01
	ERA5-Land	13.7	1.5	− 12.2	0.52	0.55	0.03
Mixed	IMERG	− 39.1	− 49.5	− 10	0.31	0.20	− 0.11
	ERA5-Land	24.9	3.7	− 21.2	0.62	0.74	0.12
Snowfall	IMERG	− 14.8	− 38.4	− 23.6	0.41	0.27	− 0.14
	ERA5-Land	66.7	20.6	− 46.1	0.22	0.59	0.37

Moreover, little has changed on the accuracies of IMERG and ERA5-Land after bias correction for rainfall. However, for mixed precipitation and snowfall, the noticeable changes happen on the accuracy of two products. The KGEʹs of IMERG decrease for both two precipitation phases while those of ERA5-Land increase. It’s worth noting that IMERG has better performance accuracy than ERA5-Land before bias correction but the contrary is the case after bias correction for snowfall. Besides, it can be seen from Table 6 that IMERG has poor performance in detecting snowfall and mixed precipitation compared with rainfall which might be ascribed to several factors^76^. First, snow accumulation on the ground often presents a similar passive microwave signature as the falling snow, making it difficult to separate them. Second, the non-spherical shape of ice particles and snowflakes results in much more complex radiative properties than the approximately spherically shaped rain drops. Third, the supercooled liquid water can increase the brightness temperature at high frequencies and obscure the brightness temperature depression signature caused by the scattering effect. Fourth, snowfall has a weaker scattering signature relative to rainfall which is more easily obscured by other signals (e.g., surface contamination and super cooled liquid water).

## Discussion

### The relationship between the change of the absolute value of RB and the change of KGEʹ after bias correction

**Figure 9:**
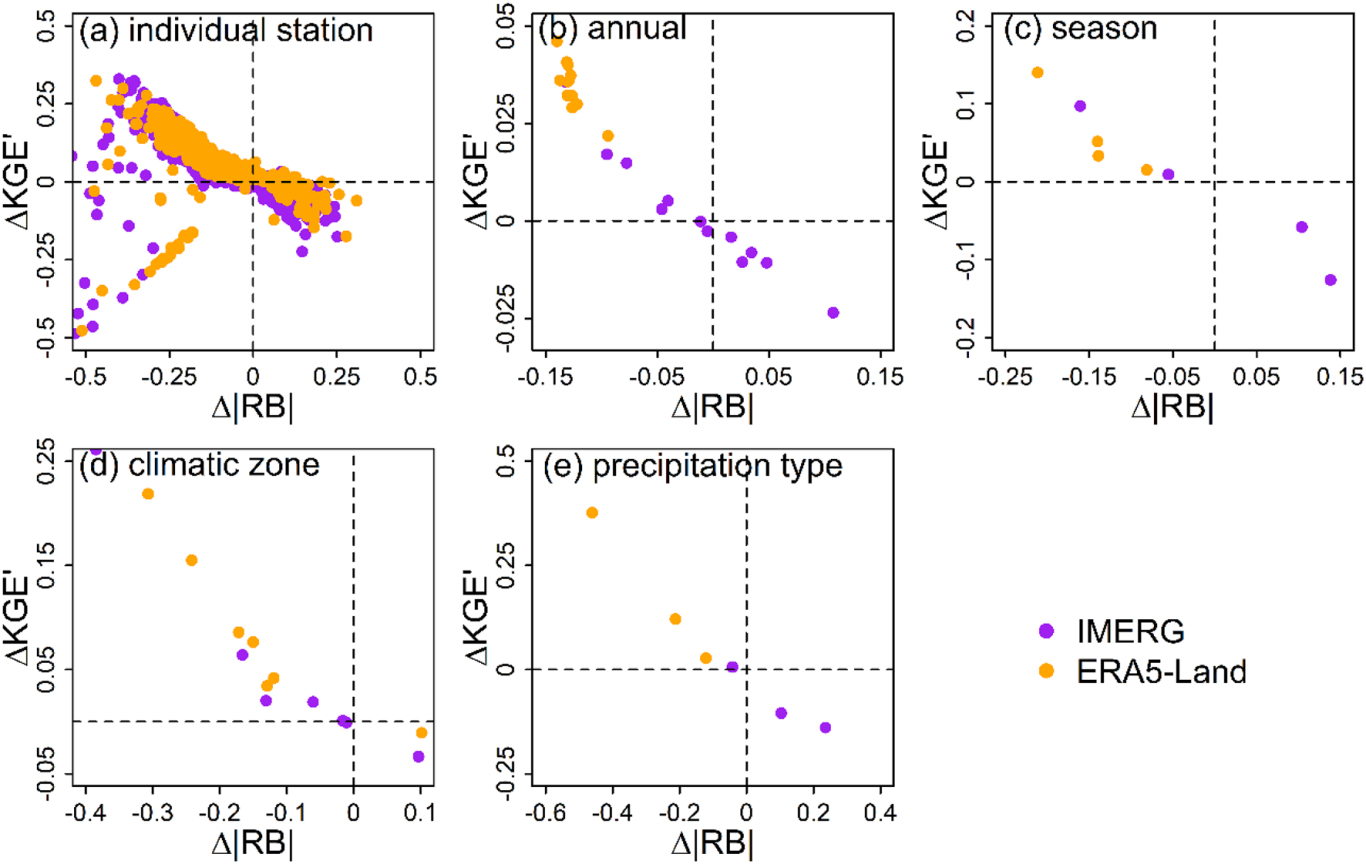
Correlations of ∆KGEʹ with ∆|RB| for IMERG and ERA5-Land at individual station scale (**a**), annual scale (**b**), seasonal scale (**c**), different climatic zones (**d**), and different precipitation phases (**e**).

After removing negative bias in gauge data, the RBs of IMERG and ERA5-Land both decline and the magnitude of RB decline is seemingly positive related to the magnitude of correction factor. Meanwhile, the KGEʹ of the two products also have changed. It seems like that the change of KGEʹ is related to the change of the absolute value of RB. We defined the absolute value of RB as |RB| and the change of |RB| as ∆|RB|. ∆|RB| is calculated by |RB| after bias correction (|RB_C|) minus |RB| before bias correction (|RB_O|). To test the preceding guess, we calculated ∆|RB| and ∆KGEʹ for IMERG and ERA5-Land at each weather station, year, season, climatic zone, and precipitation phase. The results are shown in Fig. 9.

It is evident from Fig. 9 that most of the points fall into the second quadrant or the fourth quadrant. They are along a diagonal line with the slope less than 0 in Fig. 9a–e. The sign of ∆|RB| is opposite to that of ∆KGEʹ. The magnitude of ∆|RB| is positively correlated with that of ∆KGEʹ for both IMERG and ERA5-Land. In other words, the improvement and deterioration of accuracy (KGEʹ) is consistent with the decreasing and increasing of magnitude of deviation (|RB|). The magnitude of change of accuracy is positively correlated with the magnitude of change of magnitude of deviation. This may be caused by the magnitude of deviation, which also can be represented by the term ${\left(\beta -1\right)}^{2}$ in Eq. (2), has been integrated into the calculating formula of KGEʹ.

### The strengths and weaknesses of IMERG and ERA5-Land

In this study, we have assessed the performance of IMERG and ERA5-Land. The strengths and weaknesses of IMERG and ERA5-Land whether before bias correction or after bias correction are revealed, which can be summarized as follows. ERA5-Land performs better than IMERG in northeastern China and vice versa in southeastern China. On the seasonal scale, the accuracy of IMERG is better than ERA5 in summer, while the contrary is the case in autumn and winter. In different climatic zones, the performance of IMERG is superior to ERA5-Land from severe-humid to arid climatic zone but turns into inferior to ERA5-Land in the severe-arid climatic zone. In different classes of precipitation events, the detection ability of IMERG is lower than ERA5-Land for light and moderate precipitation events but higher than ERA5-Land for extreme precipitation events. In different precipitation phases, IMERG has an advantage over ERA5-Land in rain estimation. In mixed precipitation estimation, the accuracy of IMERG is worse than that of ERA5-Land. However, in snow estimation, if directly using un-corrected gauge data, the accuracy of IMERG is significantly overvalued and that of ERA5-Land is markedly undervalued, which leads to the better performance of IMERG than ERA5-Land before bias correction but the contrary case after bias correction. The results above maybe give us insight into the strengths and weaknesses of other satellite and reanalysis precipitation products and assist us in selecting appropriate satellite and reanalysis precipitation products for different applications. In addition, the complementary strengths and weaknesses of IMERG and ERA5-Land enlighten us that satellite precipitation, reanalysis precipitation, and gauge precipitation may be able to be merged to generate a more accurate precipitation dataset for relevant hydrological applications.

### Limitations and uncertainties

The uneven distribution of weather stations may cause statistical errors when using gauge data in this study. In western China, rain gauges are scarce and unevenly distributed, especially in the western part of Tibet Plateau where rain gauges are few and even missing. Moreover, in the mountains of western China, precipitation is mainly affected by orographic uplift. Due to the lack of adequate rain gauges there, precipitation may be underestimated to some extent. In this study, we adopted a pixel-to-point strategy with the assumption that the point-scale precipitation observation is equal to the grid-scale precipitation estimates. This assumption might be justified in flat areas with a relative uniform precipitation pattern, while might not hold the regions with complex topography where precipitation has highly spatial heterogeneity.

The bias correction method may have other uncertainties. The correction for wind-induced undercatch is based on the relationship between the catch ratio of CSPG and daily mean wind speed at 10 m height. However, wind speed can vary during the 24-h period of a day, and the daily mean wind speed applied to correct wind-induced undercatch in some cases may not represent the simultaneous wind speed at the time of the occurrence of precipitation. Hourly data are preferred, but high-quality hourly precipitation and wind speed are unavailable at present. The gauge intercomparison experiments using hourly data should be carried out and new correction methods are expected to be developed on the premise of available hourly data in the future. It has been suggested that the wind speed at gauge level should be used to establish equations for catch ratios. Wind speed at gauge level can be estimated from the wind speed at standard height^77^. Reducing wind at standard height to the gauge level should consider gauge exposure^64^. Gauge exposure depends on the average vertical angle of obstacles around the gauge. The station metadata about gauge exposure were not available, so gauge exposure was not accounted for in estimating wind speed at gauge height. This may introduce some uncertainties in the estimation of gauge catch ratios. Unlike the works of Li et al.^32^, Ye et al.^36^, and Zhang et al.^58^ which used two air temperature thresholds (− 2 °C and + 2 °C) to discriminate precipitation phase, this paper adopted a more accurate parameterization scheme developed by Ding et al.^60^ that accepts wet-bulb temperature, relative humidity, and elevation instead of air temperature as inputs. However, the employed scheme doesn’t consider the temperature profile and other geophysical variables. Sims and Liu^61^ found surface skin temperature and vertical temperature lapse rate also affect the precipitation phase. The calculation of vertical temperature lapse rate needs temperature profile information, which can be obtained by observations from radiosondes and balloons. However, these data are unavailable in the sites of weather stations used in this study. On the premise of relevant data available in the future, it is expected to develop a parameterization scheme with considering the temperature profile and other geophysical variables over China in addition to the variables used by the adopted parameterization scheme in this study.

## Conclusion

This study has investigated the influence of systematic errors in gauge data on the evaluation of IMERG and ERA5-Land over China. With the removed negative bias in gauge data, the updated dataset of the gauge is used as a reference for the performance evaluation of IMERG and ERA5-Land. Comparing the evaluation results with those using original gauge data as a reference, some significant changes are found as follows:

1. The relative biases of IMERG and ERA5-Land decrease and vary in different spatial locations, years, seasons, climatic zones, and precipitation phases, which results in the change of magnitude of deviation and the reverse of the direction of deviation, i.e., the magnitude of overestimation decreases, the magnitude of underestimation increases, and the direction of deviation changes from overestimation to underestimation.
2. The frequency biases of IMERG and ERA5-Land rise in no precipitation events and decline in light, moderate, heavy, and extreme precipitation events. This is caused by the amount of gauge-observed no precipitation events significantly decreasing and the amounts of gauge-observed other four classes of precipitation events significantly increasing.
3. The change of the accuracy of two products occurs and also varies with different locations, years, seasons, climatic zones, and precipitation phases. The improvement (deterioration) of accuracy is accompanied by the decreasing (increasing) magnitude of deviation. Moreover, the magnitude of change of accuracy is positively correlated with the magnitude of change of magnitude of deviation.
4. The detection ability of two products is noticeably affected in no and light precipitation events. In no precipitation events, IMERG has better detection ability than ERA5-Land before bias correction but the detection abilities of two products become comparable after bias correction. In detecting light precipitation events, ERA5-Land has an advantage over IMERG before bias correction and the detection ability gap between them becomes bigger after bias correction.

## Data Availability

IMERG data can be downloaded from GES DISC (https://disc.sci.gsfc.nasa.gov/). ERA5-Land data are available from the ECMWF, please visit: https://cds.climate.copernicus.eu. Due to the strict security requirements from CMA, gauge data and other ground observation data used in this study are proprietary or confidential in nature. If someone wants to request these data, he or she should contact National Meteorological Science Data Center via email datacenter@cma.gov.cn.
